# Mesoporous Silica Nanoparticles Trigger Liver and Kidney Injury and Fibrosis Via Altering TLR4/NF-κB, JAK2/STAT3 and Nrf2/HO-1 Signaling in Rats

**DOI:** 10.3390/biom9100528

**Published:** 2019-09-25

**Authors:** Ayman M. Mahmoud, Ekram M. Desouky, Walaa G. Hozayen, May Bin-Jumah, El-Shaymaa El-Nahass, Hanan A. Soliman, Ahmed A. Farghali

**Affiliations:** 1 Physiology Division, Zoology Department, Faculty of Science, Beni-Suef University, Beni-Suef 62514, Egypt; 2 Biochemistry Department, Faculty of Science, Beni-Suef University, Beni-Suef 62514, Egyptwalaahozayen@hotmail.com (W.G.H.); hanan_abdelhameid@yahoo.com (H.A.S.); 3 Department of Biology, College of Science, Princess Nourah bint Abdulrahman University, Riyadh 84428, Saudi Arabia; may_binjumah@outlook.com; 4 Department of Pathology, Faculty of Veterinary Medicine, Beni-Suef University, Beni-Suef 62514, Egypt; shima_k81@yahoo.com; 5 Materials Science and Nanotechnology Department, Faculty of Postgraduate Studies for Advanced Sciences (PSAS), Beni-Suef University, Beni-Suef 62514, Egypt; ahmedfarghali74@yahoo.com

**Keywords:** mesoporous silica, Nrf2, oxidative stress, fibrosis, NF-κB

## Abstract

Mesoporous silica nanoparticles (MSNs) represent a promising inorganic platform for multiple biomedical applications. Previous studies have reported MSNs-induced hepatic and renal toxicity; however, the toxic mechanism remains unclear. This study aimed to investigate MSNs-induced hepatic and nephrotoxicity and test the hypothesis that altered TLR4/MyD88/NF-κB, JAK2/STAT3, and Nrf2/ARE/HO-1 signaling pathways mediate oxidative stress, inflammation, and fibrosis induced by MSNs. Rats were administered 25, 50, 100, and 200 mg/kg MSNs for 30 days, and samples were collected for analyses. MSNs induced functional and histologic alterations, increased the levels of reactive oxygen species (ROS), lipid peroxidation and nitric oxide, suppressed antioxidants, and Nrf2/HO-1 signaling in the liver and kidney of rats. MSNs up-regulated the expression of liver and kidney TLR4, MyD88, NF-κB p65, and caspase-3 and increased serum pro-inflammatory cytokines. In addition, MSNs activated the JAK2/STAT3 signaling pathway, down-regulated peroxisome proliferator activated receptor gamma (PPARγ), and promoted fibrosis evidenced by the increased collagen expression and deposition. In conclusion, this study conferred novel information on the role of ROS and deregulated TLR4/MyD88/NF-κB, JAK2/STAT3, PPARγ, and Nrf2/ARE/HO-1 signaling pathways in MSNs hepatic and nephrotoxicity. These findings provide experimental evidence for further studies employing genetic and pharmacological strategies to evaluate the safety of MSNs for their use in nanomedicine.

## 1. Introduction

Nanotechnology has emerged as a novel approach to the development and application of nanostructured materials in both industry and medicine [1]. Silica nanoparticles (SiNPs) possess distinct physicochemical characteristics and are now widely used in different fields [2]. The massive industrial production and global commercialization have raised the risk of human exposure to SiNPs [3]. In this context, the food additive E551 is a silicon dioxide in the nano-size range [4], and hence, the general population is exposed to it [3]. Recently, the European Food Safety Authority panel recommended a reevaluation of E551 as a food additive [5]. In addition, the use of NPs in disease diagnosis and treatment can intentionally expose humans to SiNPs [6].

Mesoporous SiNPs (MSNs) represent a promising inorganic platform for multiple applications. Given their unique characteristics, including large pore volume, high surface area, inherent biodegradability, and chemical stability, MSNs have been employed in a variety of fields [7]. MSNs have recently attracted substantial attention for use in biomedical applications, such as bioimaging, targeted drug delivery, biosensing, and others [8,9]. In addition to their unique properties, MSNs can protect their cargos from premature release and degradation [10]. Therefore, MSNs represent a promising vehicle for drug delivery. However, their toxic effects might hinder their applications in biology, medicine, and industry. The lack of data on the toxicological profile of MSNs is a major challenge to the use of MSNs in drug delivery [11]. Thus, studies are needed to evaluate the potentially toxic effects of MSNs and to explore the underlying mechanisms, particularly for improving the performance of nanomedicine. 

The growing global exposure to SiNPs raised a concern regarding their impact on human safety and health [3]. In this context, several in vitro and in vivo studies have been conducted to assess the toxic potential of SiNPs (reviewed in [3,12]). In addition, SiNPs have been shown to be as reactive as crystalline silica which is associated with silicosis, emphysema, and lung cancer [13]. Most of the studies have pointed to the major role of reactive oxygen species (ROS) in SiNPs toxicity. In vivo, MSNs induced oxidative stress in rodents as evidenced by increased lipid peroxidation and diminished antioxidants in the heart, lung [14], and liver [15]. ROS can activate nuclear factor-kappaB (NF-κB) and subsequently, the production of inflammatory mediators. Therefore, inflammation may play a substantial role in the toxicity of SiNPs. Accordingly, SiNPs elicited an inflammatory response in macrophages and the kidney of mice [16,17]. In addition to oxidative stress and inflammation, recent studies have reported SiNPs-induced hepatic [15] and renal fibrosis [17] in mice. Although these studies pointed to the involvement of transforming growth factor (TGF)-β1/Smad3 signaling in mediating SiNPs-induced fibrogenesis, other mechanisms seem to be implicated. Therefore, we explored the potential hepatic toxicity and nephrotoxicity of MSNs with an emphasis on oxidative stress, inflammation, and fibrosis, and the underlying mechanisms. We investigated the dose-dependent effect of MSNs on ROS production and nuclear factor erythroid 2-related factor 2 (Nrf2) /heme oxygenase 1 (HO-1), a signaling pathway that mainly regulates the expression of antioxidant enzymes and inhibits oxidative stress and inflammation [18]. To explore the mechanism underlying MSNs-induced inflammation and fibrosis, we scrutinized the possible involvement of toll-like receptor 4 (TLR4)/MyD88 and Janus kinase 2/signal transducer and activator of transcription 3 (JAK2/ STAT3) signaling. TLR4 contributes to NF-κB activation, and the release of inflammatory mediators [19] and persistent activation of STAT3 has been implicated in the progression of fibrosis [20,21]. Furthermore, our study pointed to the impact of MSNs on the expression of peroxisome proliferator activated receptor gamma (PPARγ), a critical regulator of adipocyte differentiation and glucose homeostasis, that suppresses NF-κB-mediated pro-inflammatory responses [22] and attenuates fibrosis [23,24].

## 2. Materials and Methods 

### 2.1. Synthesis and Characterization of MSNs

MSNs were prepared and characterized as previously described (Supplementary material) [14,25].

### 2.2. Animals and Treatments

Eight-week-old male Wistar rats, weighing 140–160 g, were obtained from VACSERA (Giza, Egypt) and housed in standard cages for one week before the start of the experiment. The animals were supplied a standard chow diet and water ad libitum and maintained on a 12 h light/dark cycle at normal temperature (23 ± 2 °C) and humidity (50–60%). The experimental protocol and all animal procedures were approved by the Institutional Animal Ethics Committee of Beni-Suef University (Egypt).

Thirty rats were divided randomly into five groups (*n* = 6). One group was kept as control and received a single intraperitoneal (ip) injection of 0.9% saline daily for 30 days. The other groups received different ip doses of MSNs (25, 50, 100 and 200 mg/kg) daily for 30 days. MSNs were suspended in 0.9% saline and frequently sonicated to avoid aggregation and sedimentation. During the experiment, mortality and clinical manifestations were reported.

### 2.3. Collection and Preparation of Samples

The animals were fasted overnight and were sacrificed under anesthesia. Blood samples were collected to prepare serum for the assay of liver and kidney function markers, interleukin (IL)-1β, IL-6, and tumor necrosis factor (TNF)-α. The animals were dissected immediately, and the liver and kidney were excised and washed in cold phosphate-buffered saline (PBS). Samples from the kidney and liver were fixed in 10% neutral buffered formalin (NBF) or kept at −80 °C. Other samples were homogenized (10% *w/v*) in cold PBS. After centrifugation of the homogenate, the clear supernatant was collected for analysis.

### 2.4. Assay of Liver and Kidney Function Markers and Pro-Inflammatory Cytokines

Serum levels of alanine aminotransferase (ALT), aspartate aminotransferase (AST), alkaline phosphatase (ALP), bilirubin, albumin, creatinine, and urea were determined using reagent kits supplied by Spinreact (Girona, Spain). IL-1β, IL-6, and TNF-α were measured in serum samples using ELISA kits (R&D Systems, Minneapolis, MN, USA).

### 2.5. Assay of Oxidative Stress Markers and Antioxidants

The level of ROS was determined in the homogenate samples using 2′,7′-dichlorodihydrofluorescein diacetate, as previously described [14,26]. Malondialdehyde (MDA), nitric oxide (NO), reduced glutathione (GSH), superoxide dismutase (SOD), catalase (CAT), and glutathione peroxidase (GPx) were determined using commercially available kits.

### 2.6. Gene Expression

Quantitative reverse transcriptase real-time polymerase chain reaction (qRT-PCR) was used to determine the effect of MSNs on the expression of collagen in the liver and kidney of rats. Briefly, RNA was isolated from the frozen samples using TRIzol (Invitrogen, Waltham, MA, USA) and was quantified using a nanodrop. RNA samples with A260/A280 ≥1.8 were selected. The integrity of RNA samples was confirmed using formaldehyde agarose gel. One µg of RNA was reverse transcribed into cDNA, which was then amplified using SYBR Green and the set of primers listed in Table 1. The obtained amplification data were analyzed using the 2^−ΔΔCt^ method [27] and normalized to β-actin.

### 2.7. Western Blotting

The frozen liver and kidney samples were homogenized in radioimmunoprecipitation assay (RIPA) buffer supplemented with proteinase inhibitors. The homogenate was centrifuged at 10000 rpm, and the supernatant was collected, and protein concentration was determined using the Bradford protein assay kit (BioBasic, Markham, Canada). Forty µg of protein from each sample was electrophoresed using 10% sodium dodecyl sulfate-polyacrylamide gel electrophoresis (SDS-PAGE) and transferred to nitrocellulose membranes. The membranes were blocked with 5% skimmed milk in Tris-buffered saline/Tween 20 (TBST) for 1 h at room temperature (RT). The blocked membranes were probed with primary antibodies against TLR4, MyD88, NF-κB p65, p-JAK2, JAK2, p-STAT3, STAT3, suppressor of cytokine signaling 3 (SOCS3), cleaved caspase-3, PPARγ, Nrf2, HO-1, and glyceraldehyde-3-phosphate dehydrogenase (GAPDH) (Novus Biologicals, Centennial, CO, USA) overnight at 4 °C. After washing with TBST, the membranes were incubated with secondary antibodies and developed by an enhanced chemiluminescence kit (BIO-RAD, Hercules, CA, USA). The bands were quantified by ImageJ (version 1.32j, NIH, USA) [28], normalized to GAPDH and presented as a percent of the control.

### 2.8. Histological Examination

The liver and kidney samples collected on 10% NBF were fixed for 48 h. The tissues were processed via routine histological procedures, and 5-μm sections were cut using a microtome. For examination and scoring the histological alterations, the sections were stained with hematoxylin and eosin (H&E), whereas Masson’s trichrome (MT) staining was used to evaluate hepatic and renal collagen deposition. The MT-positive areas were quantified using ImageJ.

### 2.9. Statistical Analysis

The obtained data were analyzed using GraphPad Prism 7 (GraphPad Software, San Diego, CA, USA) and expressed as mean ± standard error of the mean (SEM). All statistical comparisons were performed by one-way ANOVA test followed by Tukey’s test. The difference was considered significant at *p-*value less than 0.05.

## 3. Results

### 3.1. Characterization of MSNs

**Figure 1:**
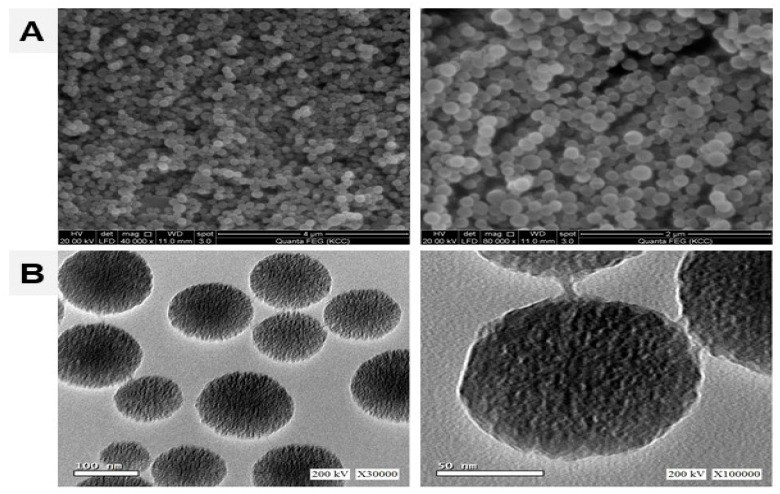
Scanning electron microscopy (SEM) (**A**) transmission electron microscopy (TEM) (**B**) of mesoporus silica nanoparticles (MSNs) showing spherical, non-aggregated, and well-dispersed particles with uniform size.

Both the scanning and transmission electron microscopy (SEM and TEM) examination of MSNs revealed spherical, non-aggregated, and well-dispersed particles with uniform size (~50 nm) and uniformly scattered dendrimeric fibers (Figure 1). In addition, we have recently characterized the prepared MSNs using dynamic light scattering (DLS), zeta potential measurement, thermogravimetric analysis (TGA)/Differential scanning calorimetry (DSC) and X-ray diffraction (XRD) as represented in Supplementary Figure S1 [14].

### 3.2. MSNs Induce Hepatic and Nephrotoxicity in Rats

To evaluate the effect of MSNs on liver and kidney of rats, we measured liver and kidney function markers and performed a histologic examination. Rats received MSNs showed a significant (*p* < 0.001) increase in serum ALT, AST, and ALP when compared with the control group (Table 2). Serum bilirubin was remarkably increased in rats received 25 (*p* < 0.05), 50 (*p* < 0.05), 100 (*p* < 0.05) and 200 mg/kg (*p* < 0.001) MSNs as represented in Table 2. In contrast, serum albumin was decreased in rats received 50 (*p* < 0.05), 100 (*p* < 0.01), and 200 mg/kg (*p* < 0.001) MSNs, while showed non-significant changes at the lower dose (Table 2). Creatinine and urea were increased in 25 (*p* < 0.001; *p* < 0.01), 50 (*p* < 0.001; *p* < 0.01), 100 (*p* < 0.001; *p* < 0.01), and 200 mg/kg (*p* < 0.001; *p* < 0.001) MSNs-administered rats (Table 2).

**Figure 2:**
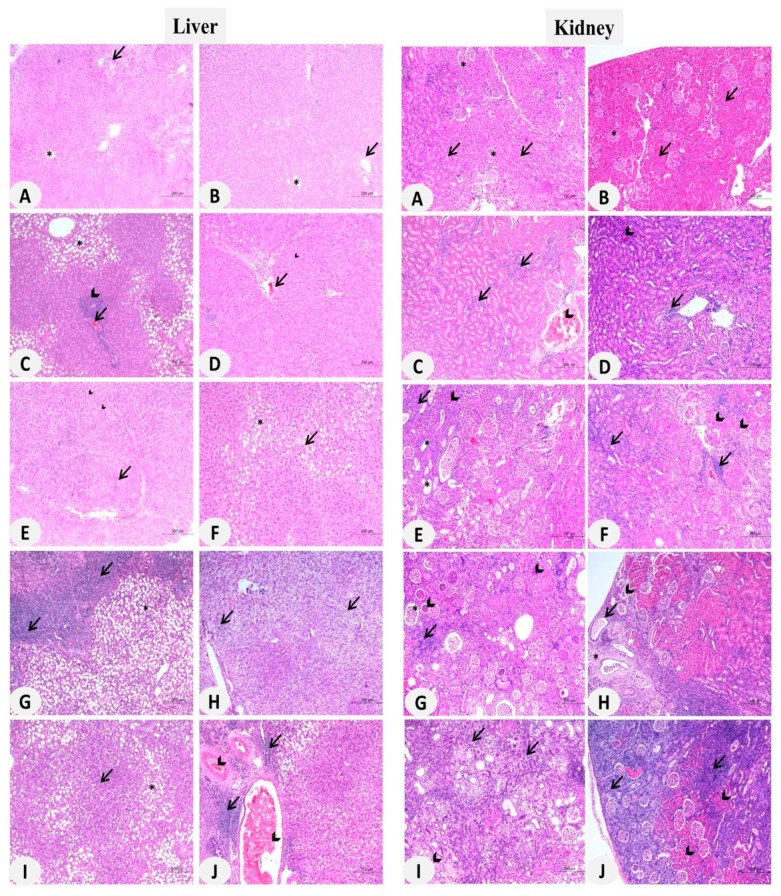
MSNs-induced histological alteration in the liver and kidney of rats. (Left panel) Photomicrographs of liver sections of (**A,B**) control group showing normal hepatic lobule, central veins (*), and portal area (arrows), (**C,D**) rats received 25 mg/kg MSNs showing moderate fatty changes of hepatocytes (*), mild leukocyte infiltration in portal areas (arrow head), congestion (arrow) (**C**), degenerative changes, proliferation of Kupffer cells (KCs) (arrow heads) and leukocyte infiltration in the portal area (arrow) (**D**), (**E,F**) rats received 50 mg/kg MSNs showing degenerative changes of hepatocytes (arrows), proliferation of KCs (arrow heads) (**E**), fatty changes of hepatocytes (*), leukocyte infiltration and congestion (arrow) (**F**), (**G,H**) rats received 100 mg/kg MSNs showing fatty changes (*), leukocyte infiltration (arrows) (**G**), and vacuolar degeneration (arrows) (**G**), and (**I,J**) rats received 200 mg/kg MSNs showing fatty changes (*), necrosis (arrows) (**I**), leukocyte infiltration (arrows) and congestion (arrow heads) (**J**). (Right panel) Photomicrographs of kidney of (**A,B**) control group showing normal glomeruli (*) and renal tubules (arrow head), (**C,D**) rats received 25 mg/kg MSNs showing interstitial inflammatory cell aggregates (arrows), congestion (arrow head) (**C**), perivascular inflammatory cell aggregates (arrows) and congestion (arrow head) (**D**), (**E,F**) rats received 50 mg/kg MSNs showing inflammatory cell reaction (arrows), dilation of some renal tubules (*), congestion (arrow head) (**E**), inflammatory cell reactions (arrows) and necrosis (arrow head) (**F**), (**G,H**) rats received 100 mg/kg MSNs showing inflammatory cell aggregates (arrows), dilatation of Bowman’s space (*), glomerular atrophy (arrow heads) (**G**), proliferation of fibrous connective tissues (*), dilation of renal tubules (arrow), and necrosis (**H**) and (**I,J**) rats received 200 mg/kg MSNs showing necrotic changes (arrows), proliferation of interstitial fibrous connective tissues (arrow head) (**I**), lymphocytic interstitial nephritis (arrows) and coagulative necrosis (arrow heads) (**J**). (hematoxylin-eosin, H&E, X100).

The hepatic and nephrotoxic effects of MSNs were confirmed by the histopathological findings. H&E-stained sections in the liver (Figure 1; Left panel) of control group revealed the normal structure of the hepatic lobules, hepatocytes, and sinusoids (Figure 2A–B). The liver of animals received 25 mg/kg (Figure 2C–D) and 50 mg/kg (Figure 2E–F) MSNs showed multiple histological alterations, including degenerative changes, necrosis, leukocyte infiltration in the portal area, fatty changes, congestion and Kupffer cells (KCs) proliferation. In addition to these alterations, rats received 100 mg/kg and 200 mg/kg MSNs exhibited granulomatous reactions as showed in Figure 2G–J, respectively, and Table 3.

**Figure 3:**
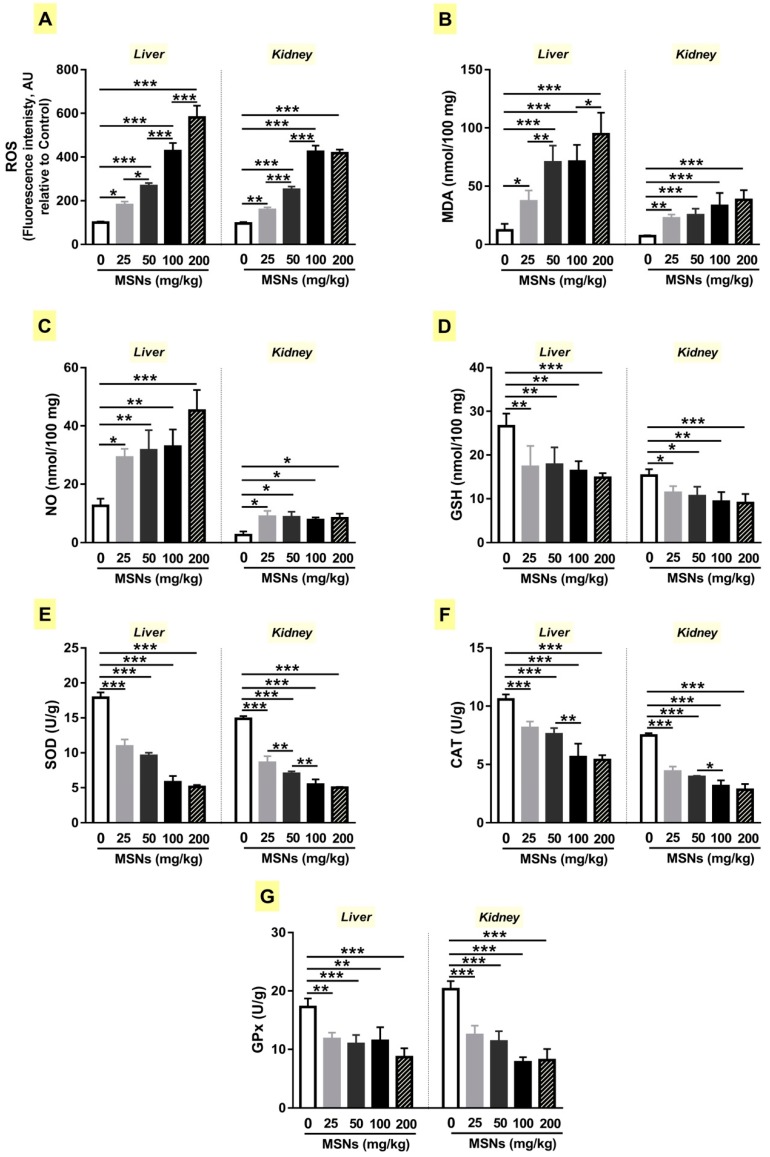
MSNs provoke reactive oxygen species (ROS) production and oxidative stress in liver and kidney of rats. MSNs increased ROS (**A**), malondialdehyde (MDA) (**B**) and nitric oxide (NO) (**C**), and diminished reduced glutathione (GSH) (**D**), superoxide dismutase (SOD) (**E**), catalase (CAT), (**F**) and glutathione peroxidase (GPx) (**G**). Data are Mean ± SEM (*n* = 6). *p < 0.05, **p < 0.01, and ***p < 0.001.

Normal histological architecture of the capsule, cortex, medulla, renal tubules, and glomeruli was observed in the kidney sections of control rats (Figure 3A,B, Right panel). However, rats received the lower dose of MSNs showed tubular degeneration, glomerulonephritis, glomerular hypercellularity, leukocyte infiltration and chronic nephritis (Figure 3C,D). In addition to these manifestations, tubular necrosis was noticed in rats received 50 mg/kg MSNs (Figure 3E,F), whereas the 100 (Figure 3G,H) and 200 mg/kg (Figure 3I,J) MSNs induced glomerular atrophy, tubular dilatations, casts, cystic dilatation, and edema. Dilatation of Bowman’s capsule was observed in the kidney of rats administered with 200 mg/kg MSNs (Table 3).

### 3.3. MSNs Provoke ROS Production and Oxidative Stress in the Liver and Kidney of Rats

ROS, MDA, NO, and antioxidant defenses were determined to evaluate the effect of MSNs on the redox balance in the liver and kidney of rats. ROS were significantly increased in the liver and kidney of rats received 25 (*p* < 0.05; *p* < 0.01), 50 (*p* < 0.5; *p* < 0.001), 100 (*p* < 0.001; *p* < 0.001), and 200 mg/kg (*p* < 0.001; *p* < 0.001) MSNs (Figure 3A). MDA was remarkably increased in the liver and kidney of rats following chronic administration of MSNs (Figure 3B). NO was notably increased in the liver and kidney of rats following the administration of MSNs (Figure 3C).

GSH was significantly declined in the liver and kidney of rats that received different doses of MSNs (Figure 3D). SOD and CAT showed a significant (*p* < 0.001) decrease in the liver and kidney of rats administered MSNs (Figure 3E,F, respectively). GPx activity was declined significantly in rats received all doses of MSNs (Figure 3G).

### 3.4. MSNs Suppress PPARγ Expression and Nrf2/HO-1 Signaling in Liver and Kidney of Rats

**Figure 4:**
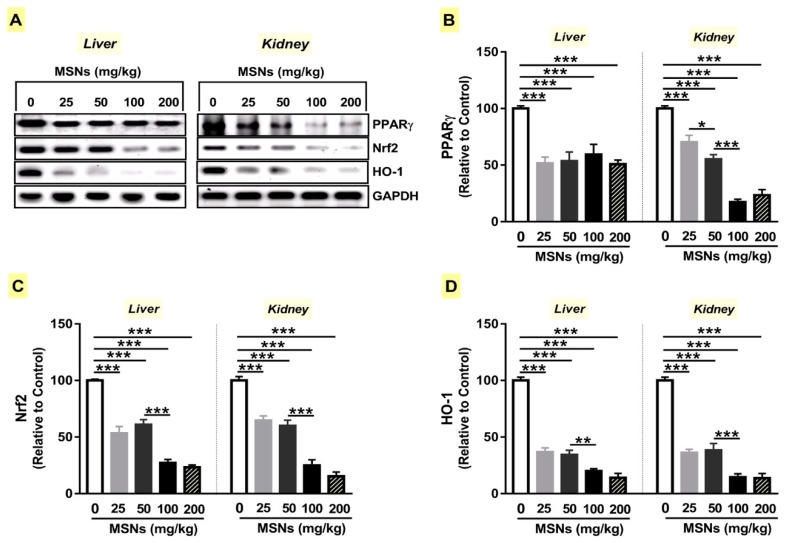
MSNs suppress peroxisome proliferator activated receptor gamma (PPARγ) expression and nuclear factor erythroid 2-related factor 2 (Nrf2) /heme oxygenase 1 (HO-1) signaling in the liver and kidney of rats. (**A**) Representative blots of PPARγ, Nrf2, and HO-1. (**B**–**D**) The expression of PPARγ (**B**), Nrf2 (**C**), and HO-1 (**D**) was significantly decreased in the liver and kidney of MSNs-induced rats. Data are mean ± SEM (*n* = 6). *p < 0.05, **p < 0.01, and ***p < 0.001.

PPARγ can regulate the expression of antioxidant enzymes [29], and suppress inflammation and fibrosis [22,23]. Therefore, we determined the effect of MSNs on PPARγ expression by western blotting. MSNs suppressed the protein expression levels of PPARγ in the liver and kidney of rats (*p* < 0.001; Figure 4A,B). The 50 mg/kg MSNs down-regulated kidney PPARγ significantly (*p* < 0.05) when compared with the lower dose. In addition, the 100 mg/kg markedly down-regulated PPARγ expression in the kidney of rats received lower doses.

To explore the effect of MSNs on Nrf2 signaling, we determined the protein levels of Nrf2 and HO-1 in the liver and kidney of rats. MSNs reduced Nrf2 and HO-1 expression significantly (*p* < 0.001; *p* < 0.001; Figure 4A,C,D) in both the liver and kidney. When compared with the lower dose, the 100 mg/kg MSNs down-regulated the liver and kidney Nrf2 (*p* < 0.001; *p* < 0.001) and HO-1 (*p* < 0.01; *p* < 0.001). 

### 3.5. MSNs Activate TLR4/MyD88/NF-κB Signaling and Apoptosis in the Liver and Kidney of Rats

**Figure 5:**
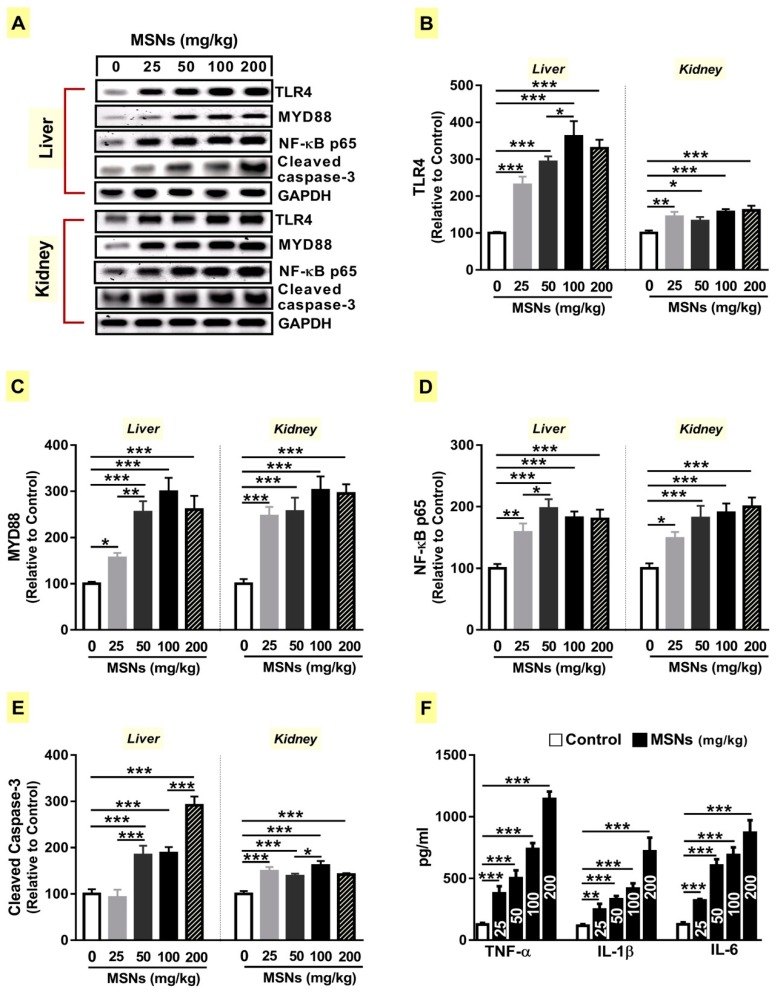
MSNs activate TLR4/MyD88/NF-κB signaling and apoptosis in the liver and kidney of rats. (**A**) Representative blots of TLR4, MyD88, NF-κB p65, and cleaved caspase-3. MSNs up-regulated TLR4 (**B**), MyD88 (**C**), NF-κB p65 (**D**), and cleaved caspase-3 (**E**), and increased levels of serum TNF-α, IL-1β, and IL-6 (**F**). Data are mean ± SEM (*n* = 6). *p < 0.05, **p < 0.01, and ***p < 0.001.

Given the role of TLR4 activation and its downstream adapter MyD88 in activating the NF-κB signaling pathway [19], we examined the effects of MSNs on TLR4/MyD88/NF-κB signaling. Rats received the lower dose of MSNs exhibited increased expression of TLR4 (*p* < 0.001; Figure 5A,B), MyD88 (*p* < 0.05; Figure 5A,C) and NF-κB p65 (*p* < 0.01; Figure 5A,D). The higher doses increased the protein expression levels of TLR4 (Figure 5B), MYD88 (Figure 5C), and NF-κB p65 (Figure 5D) significantly (*p* < 0.001) in the liver of rats. In the kidney, MSNs activated TLR4/MyD88/NF-κB signaling as revealed by the increased expression of TLR4 (Figure 5A,B), MyD88 (Figure 5A,C), and NF-κB p65 (Figure 5A,D) when administered at the doses of 25 (*p* < 0.01; *p* < 0.001; *p* < 0.05), 50 (*p* < 0.05; *p* < 0.001; *p* < 0.001), 100 (*p* < 0.001; *p* < 0.001; *p* < 0.001), and 200 mg/kg (*p* < 0.001; *p* < 0.001; *p* < 0.001).

Next, we determined serum levels of pro-inflammatory cytokines. All doses of MSNs increased (*p* < 0.001) the levels of TNF-α, IL-1β, and IL-6 (Figure 5F).

The apoptosis marker caspase-3 showed a significant increase in the liver and kidney of 50, 100, and 200 mg/kg MSNs-administered rats (Figure 5A,E). The lower MSNs dose (25 mg/kg) did not activate liver caspase-3 (*p* > 0.05); however, caspase-3 was significantly (*p* < 0.001) activated in the kidney of rats receiving the same dose.

### 3.6. MSNs Induce Liver and Kidney Fibrosis in Rats

**Figure 6:**
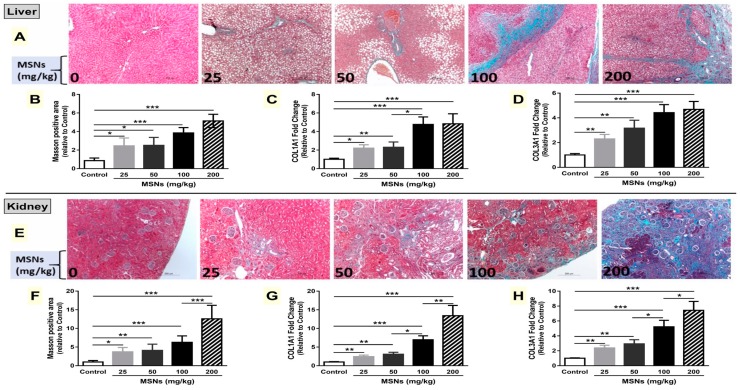
MSNs induce liver and kidney fibrosis in rats. (**A**,**E**) Photomicrographs of Masson’s trichrome-stained liver (**A**) and kidney (**E**) (X100). MSNs induced a significant increase in the deposition of collagen (**B**,**F**) and expression of collagen I (**C**,**G**) and collagen III (**D**,**H**) in the liver and kidney. Data are mean ± SEM (*n* = 6). *p < 0.05, **p < 0.01, and ***p < 0.001.

Microscopic examination revealed an increase in the deposition of extracellular matrix (ECM) in the liver (Figure 6A,B) and kidney (Figure 6E,F) of MSNs-induced rats. mRNA expression of collagen I and III was significantly increased in the liver (Figure 6C,D) and kidney (Figure 6G,H) of MSNs-induced rats.

### 3.7. MSNs Activate JAk2/STAT3 Signaling in the Liver and Kidney of Rats

The pro-inflammatory mediators, notably IL-6, produced as a result of NF-κB activation could bind to its heterodimeric receptor (IL-6Rα and IL-6Rβ (gp130)), resulting in transphosphorylation and activation of JAK2. Activated JAK2 phosphorylates STAT3, which homodimerizes and accumulates in the nucleus where it controls the expression of multiple genes. The persistent activation of STAT3 is associated with chronic inflammation and fibrosis [20,21]. Therefore, we investigated the changes in JA2/STAT3 signaling in MSNs-administered rats.

**Figure 7:**
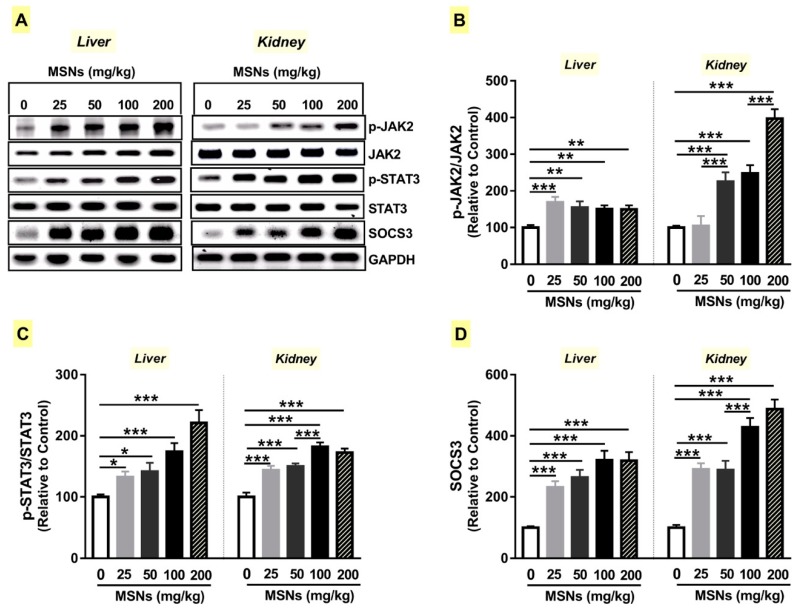
MSNs activate Janus kinase 2/signal transducer and activator of transcription 3 (JAk2/STAT3) signaling in liver and kidney of rats. (**A**) Representative blots of p-JAK2, JAK2, p-STAT3, STAT3 and SOCS3. MSNs increased phosphorylation levels of JAK2 (**B**) and STAT3 (**C**), and the expression of SOCS3 (**D**). Data are mean ± SEM (*n* = 6). *p < 0.05, **p < 0.01, and ***p < 0.001.

MSNs induced a significant increase in JAK2 and STAT3 phosphorylation and SOCS3 expression (Figure 7A–D) in the liver of rats when administered at doses of 25 (*p* < 0.001; *p* < 0.05; *p* < 0.001), 50 (*p* < 0.01; *p* < 0.05; *p* < 0.001), 100 (*p* < 0.01; *p* < 0.001; *p* < 0.001), and 200 mg/kg (*p* < 0.01; *p* < 0.001; *p* < 0.001).

In the kidney of MSNs-induced rats, the effect of the 25 mg/kg dose on JAK2 phosphorylation was non-significant (*p* > 0.05). However, the same dose significantly increased the phosphorylation of STAT3 (*p* < 0.001) and expression of SOCS3 (*p <* 0.001), as represented in Figure 7A–D. The higher doses induced remarkable (*p* < 0.001) increase in JAK2 and STAT3 phosphorylation and SOCS3 expression in the kidney of rats (Figure 7A–D).

## 4. Discussion

Studies are needed to investigate the mechanisms implicated in the toxicity of MSNs to improve their biomedical applications. In this study, we investigated the mechanisms underlying the hepatic and renal toxicity of MSNs in vivo. MSNs induced liver injury evidenced by the dose-dependent increase in ALT, AST, ALP, and bilirubin, along with decreased albumin. Given its role in the recognition and clearance of NPs [30], the liver is vulnerable to SiNPs-induced injury. This notion is supported by studies showing that the largest holder for intravenously administered SiNPs is the liver [31], and the dose-dependent increase in serum ALT in a mouse model of SiNPs-induced acute toxicity [32]. In addition, mice that received 30 mg/kg SiNPs (70 nm) exhibited liver injury marked by increased serum ALT and histological alterations, with no effect on the kidney [33]. In contrast, mice that received intragastric MSNs (40 mg/kg) showed normal liver function, whereas creatinine was significantly elevated at day 14 post-administration [34]. Recently, Yu et al. [15] demonstrated elevated serum levels of aminotransferases and decreased albumin in mice receiving 20 mg/kg amorphous silica NPs, once every three days for 15 days. MSNs-induced liver injury in our study was proved by the histological findings, including degenerative changes, necrosis, fatty changes, congestion, KCs proliferation, leukocyte infiltration in the portal area, and granulomatous reactions. In support of these findings, a recent study demonstrated hydropic degeneration, apoptosis, leukocyte infiltration, central vein hyperemia, and granulomas in mice that received 20 mg/kg amorphous SiNPs at days 15, 30, and 60 after administration [15]. Moreover, MSNs-induced rats showed nephrotoxicity manifested by the significantly elevated serum creatinine and urea. Accordingly, mice showed increased serum creatinine at day 14 after intragastric administration of 40 mg/kg MSNs; however, liver function markers were not affected [34]. Histological examination revealed tubular degeneration, glomerulonephritis, glomerular hypercellularity, leukocyte infiltration, and chronic nephritis in 25 mg/kg MSNs-administered rats. In addition to these manifestations, tubular necrosis was noticed in rats that received 50 mg/kg MSNs, whereas the higher doses induced glomerular atrophy, tubular dilatation, casts, and cystic, and Bowman’s capsule dilatation. In support of these findings, a single ip injection of 300 and 600 mg/kg MSNs induced lymphocytic infiltration, renal tubular regeneration, and interstitial fibrosis in mice at day 12 post-injection [17]. Our findings pointed to the potential of MSNs to induce liver and kidney functional and structural alterations. However, the mechanisms underlying the toxicity of MSNs are not fully understood.

Recent studies have shown that both oxidative stress and inflammation are implicated in the toxicity of MSNs. Therefore, we determined ROS, MDA, and NO levels, as well as antioxidants in the liver and kidney of MSNs-induced rats. Herein, MSNs promoted a dose-dependent increase in ROS in both the liver and kidney of rats. Excessive production of ROS could provoke lipid peroxidation and damage cellular proteins and DNA. Previous in vitro studies have shown a dose-dependent increase in ROS production in human and rat cell lines treated with SiNPs [35,36]. Recently, we demonstrated increased cardiac and pulmonary ROS in MSNs-induced rats [14]. The potential of SiNPs to induce ROS generation has been attributed to the silicon-bonded hydroxyl groups on the particle surface and the unsaturated bond [37]. As a consequence of excess ROS, MSNs-induced rats exhibited an increase in MDA and diminished GSH and activity of SOD, CAT, and GPx. These findings point to the oxidative stress status provoked by MSNs in the liver and kidney of rats. Yu et al. [15] have recently demonstrated lipid peroxidation and decreased SOD and GPx in mice livers at day 15 after the injection of 20 mg/kg amorphous SiNPs. In addition, MSNs provoked NO production in both the liver and kidney of rats which could be explained by the increased expression of inducible NO synthase (iNOS) secondary to excessive ROS generation. Previous studies showed accumulation of SiNPs in KCs [15], leading to increased ROS, NO, and expression of pro-inflammatory cytokines [38].

The mechanisms underlying SiNPs-induced production of ROS have been investigated by several authors. Increased ROS in response to SiNPs has been attributed to the intrinsic production by the particles themselves, activation of nicotinamide adenine dinucleotide phosphate (NADPH) oxidase (NOX) and mitochondrial dysfunction [17,39]. Kojima et al. [40] demonstrated increased ATP release from P2X7 receptors in 30 nm SiNPs-induced KCs in vitro, resulting in increased NOX-mediated ROS generation. In addition, the redox potential sensor transient receptor potential melastatin 2 (TRPM2) has been shown to regulate NOX activity and ROS generation in human embryonic kidney 293 cells treated with SiNPs [17]. However, studies explored the mechanism of SiNPs-induced ROS generation in vivo are scarce. Herein, we showed the involvement of Nrf2/HO-1 signaling pathway in MSNs-induced overproduction of ROS. Nrf2 is a transcription factor that controls the expression of antioxidants, including HO-1 [41]. Upon exposure to mild oxidative stress, ROS disturbs the Keap1–Cullin 3 ubiquitination system that degrades Nrf2. Consequently, Nrf2 translocate into the nucleus, complex with small MAF and bind to antioxidant response element (ARE), leading to increased expression of antioxidant genes [42]. Therefore, inactivation of Nrf2 signaling can result in diminished antioxidants and increased ROS levels. Our results showed dose-dependent suppression of Nrf2 in MSNs-administered rats. The suppressed Nrf2 signaling was confirmed by the decreased expression of HO-1 along with the diminished antioxidant enzymes in MSNs-administered rats. The suppressed expression of Nrf2 in the present study could be attributed to the excessive and sustained oxidative stress in MSNs-induced rats. This notion is supported by our recent studies where prolonged periods of drug-induced oxidative stress was associated with diminished Nrf2 signaling in the liver and kidney of rodents [43,44,45,46,47,48].

Inflammation is an important element in the evaluation of NPs toxicity. MSNs activated NF-κB and subsequently increased the release of TNF-α, IL-1β, and IL-6. NF-κB has a prominent role in inflammation and oxidative stress, and some in vitro and in vivo studies demonstrated its activation in response to SiNPs [16,17]. In addition to ROS, TLR4 activation and MyD88 play an important role in the activation of NF-κB [19]. Therefore, we thought that the TLR4/MyD88/NF-κB signaling might be implicated in MSNs-induced liver and kidney injury. Our findings showed increased expression of TLR4 and MyD88 in MSNs-administered rats. TLRs are widely expressed in different cells, including KCs, hepatocytes, and hepatic stellate cells (HSCs), and TLRs-mediated signaling has been involved in liver and kidney diseases [49,50]. Given that TLR4 could be stimulated by endotoxins, we tested our MSNs samples to exclude any endotoxin contamination. Therefore, our study introduced evidence that TLR4/MyD88/NF-κB signaling is one of the pathways implicated in MSNs-induced liver and kidney injury. Furthermore, MSNs-induced suppression of Nrf2 could be implicated in the activation of NF-κB signaling. Nrf2 diminishes oxidative stress and NF-κB [43,44]; therefore, the loss of Nrf2 function elicits oxidative stress and amplifies inflammation. In addition, overexpression of the NF-κB p65 subunit could increase the abundance of nuclear Keap1, resulting in inhibition of Nrf2 signaling [51]; however, the capability of Keap1 to deactivate Nrf2 within the nucleus is debated [52]. Besides its ability to suppresses inflammation through redox control, Nrf2 has been identified as an upstream regulator of pro-inflammatory cytokine production [53]. Nrf2 suppressed both in vitro and in vivo macrophage inflammatory response independently of the Nrf2-binding motif and ROS level [53].

Several studies have demonstrated the potential of SiNPs to induce liver fibrosis [15,54]. Here, we showed that MSNs induced fibrosis in both the liver and kidney of rats as evidenced by the MT staining and the expression of collagen. In accordance with our findings, oral exposure to 100, 500, or 1000 mg/kg nanostructured silica for 84 days up-regulated the fibrosis-related genes in the liver of rats [54]. Mice received repeated intravenous administration of 15, 30, or 60 mg/kg SiNPs showed increased deposition of collagen mediated via activation of TGF-β1/Smad3 signaling in the liver [15]. In addition, mice received an ip high dose of MSNs showed renal interstitial fibrosis mediated via activation of NF-κB signaling [17]. TGF-β is a core pathway of fibrosis, and activation of TGF-β1/Smad3 signaling increases the expression of pro-fibrotic genes [55]. We have recently reported up-regulation of TGF-β1/Smad3 signaling associated with inflammation and fibrosis in the liver of rats [56]. To explore the mechanism underlying MSNs-induced liver and kidney fibrosis in rats, we investigated the role of JAK2/STAT3 signaling. In addition to its role in inflammation, we hypothesized that STAT3 might be implicated in MSNs-induced fibrosis. Herein, MSNs promoted JAK2 and STAT3 phosphorylation in the liver and kidney of rats. Although it has an important role in cell survival and proliferation, persistent activation of STAT3 is associated with various pathological conditions, including fibrosis [20,21]. The molecular mechanisms of the involvement of STAT3 in fibrosis are not fully elucidated; however, there is evidence that STAT3 induces the production of collagen type I [57]. Our results added support to this notion where activation of JAK2/STAT3 signaling was associated with up-regulation of collagen I and III expression. Recently, STAT3 has been reported to control COL1A2 enhancer activation directly [57]. Other studies have attributed TGFβ-induced increase in collagen production to STAT3. In this context, TGF-β-mediated phosphorylation of STAT3 at S727 is involved in the fibrotic element of fibrostenotic Crohn’s disease [58]. Inhibition of STAT3 signaling in fibrotic kidney cells suppressed the expression of collagen I and other fibrosis mediators, independently of Smad3 [59]. In addition, STAT3 can control the expression of matrix metalloproteinases (MMPs) which play a key role in the maintenance of the ECM. In a model of renal fibrosis, activation of STAT3 was associated with the up-regulation of MMP-9 [60]. Furthermore, JAK2 has previously been demonstrated as a mediator of non-canonical TGFβ signaling [61]. Recently, Chakraborty et al. [62] have shown that STAT3 acts as a mediator of the pro-fibrotic effects of TGF-β. In cultured fibroblasts, siRNA-induced knockdown and pharmacological inhibition of STAT3 diminished TGFβ-induced differentiation, as well as collagen release [62]. Therefore, activation of JAK2/STAT3 signaling and subsequent collagen production contributes significantly to MSNs-induced liver and kidney fibrosis in rats.

Owing to the association between ROS, inflammatory cytokines, and apoptosis, and the growing evidence pointing to the role of apoptosis in fibrogenesis [15,63,64], we determined cleaved caspase-3 expression in MSNs-induced rats. Here, we observed an increase in liver and kidney cleaved caspase-3 in rats received different doses of MSNs. Inhibition of caspase-mediated apoptosis has been reported as an effective strategy to ameliorate murine lung fibrosis [63]. In the liver, apoptotic hepatocytes are rapidly engulfed by KCs, and the accumulation of apoptotic bodies triggers the production of TNF-α and the release of TGF-β1 from HSCs, resulting in hepatic inflammation, extended apoptosis of hepatocytes and fibrosis [64,65]. Therefore, persistent apoptosis is one of the factors implicated in MSNs-induced liver and kidney fibrosis.

PPARγ could directly activate the expression of antioxidant enzymes [29], and suppress NF-κB [22] and TGF-β1/Smad3 [23]. Therefore, we hypothesized that it might have a role in the MSNs-induced oxidative stress, inflammation, and fibrosis. Interestingly, PPARγ was down-regulated in MSNs-induced rats. Studies have shown remarkable liver fibrosis in PPARγ-deficient mice and suppressed HSCs and fibrogenesis following PPARγ overexpression [66]. PPARγ can bind Smad3 and suppress the nuclear accumulation of p-Smad3 and TGF-β1 signaling [23]. Pharmacologic activation of PPARγ inhibited dermal fibrogenesis by suppressing the early growth response protein 1, a mediator of non-Smad TGF-β1 signaling [24], and maintained normal epithelial phenotype, prevented renal fibrosis and oxidative stress in mice [67]. Our findings offer the first evidence showing the role of PPARγ in MSNs-induced liver and kidney injury. 

## 5. Conclusions

**Figure 8:**
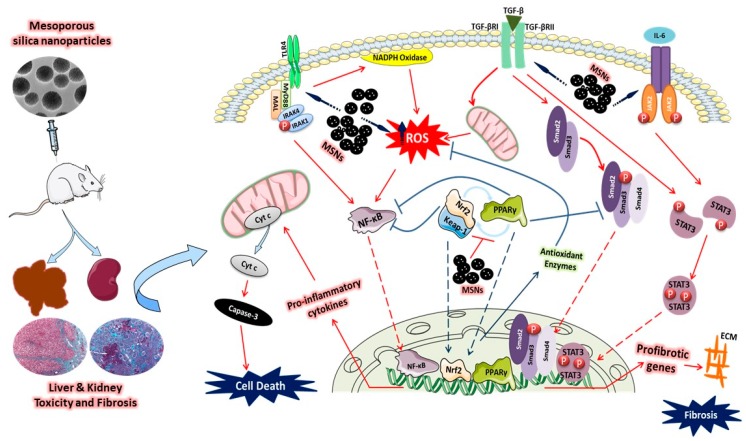
A proposed schematic diagram illustrating MSNs-induced hepatic and renal toxicity and fibrosis. MSNs provoke the production of ROS through the intrinsic production, mitochondrial dysfunction, and stimulation of NADPH oxidase, and activate the TLR4/MyD88 signaling. ROS and elicited TLR4/MyD88 signaling activates NF-κB, and subsequently the release of pro-inflammatory cytokines, resulting in apoptotic cell death via activation of caspase-3. MSNs activate both TGF-β/Smad3 and JAK2/STAT3 signaling, increase the transcription of profibrotic genes, and promote deposition of ECM and fibrosis. The increased ROS production, inflammation, and fibrosis induced by MSNs are augmented by the down-regulation of PPARγ and Nrf2/ARE/HO-1 signaling.

Our findings show that MSNs have the potential to induce a dose-dependent liver and kidney injury in rats. The impaired function of the liver and kidney was mediated via MSNs-induced oxidative stress, inflammation, fibrosis, and tissue injury. The obtained data conferred new information on the involvement of activated TLR4/MyD88/NF-κB and JAK2/STAT3 signaling and suppressed the Nrf2/ARE/HO-1 pathway in mediating the in vivo toxicity of MSNs. Furthermore, this study pointed to the ability of MSNs to suppress PPARγ expression in the liver and kidney of rats (summarized mechanistic pathways are represented in Figure 8). Together, the findings of this study enriched our understanding of the mechanisms implicated in the toxicity of MSNs. However, further studies employing genetic and pharmacological strategies are needed to delineate the exact involvement of these signaling pathways in MSNs toxicity. Understanding the exact mechanisms underlying its toxicity will provide strategies for the production of MSNs with minimum or no toxic effects and improve their biomedical application.

## Figures and Tables

**Table 1:** Primers used for qRT-PCR.

Gene	Forward Primer Sequence (5′-3′)	Reverse Primer Sequence (5′-3′)
*COL1A1*	GTACATCAGCCCAAACCCCA	CAGGATCGGAACCTTCGCTT
*COL3A1*	AGGGCAGGGAACAACTGATG	GGTCCCACATTGCACAAAGC
*β-actin*	AGGAGTACGATGAGTCCGGC	CGCAGCTCAGTAACAGTCCG

qRT-PCR: quantitative reverse transcriptase real-time polymerase chain reaction.

**Table 2:** Effect of MSNs on liver and kidney function markers in rats.

	Control	MSNs			
		25 mg/kg	50 mg/kg	100 mg/kg	200 mg/kg
ALT (U/L)	21.63 ± 1.71	82.86 ± 7.45 ***	109.71 ± 3.19 ***	111.36 ± 3.79 ***	137.07 ± 4.69 ***
AST (U/L)	47.27 ± 2.34	153.06 ± 5.09 ***	157.20 ± 13.65 ***	175.19 ± 9.87 ***	179.00 ± 4.39 ***
ALP (U/L)	166.50 ± 7.97	305.31 ± 10.74 ***	313.09 ± 8.23 ***	589.14 ± 27.16 ***	735.93 ± 25.09 ***
Bilirubin (mg/dl)	0.52 ± 0.07	1.86 ± 0.17 *	1.83 ± 0.26 *	1.82 ± 0.21 *	2.95 ± 0.42 ***
Albumin (g/dl)	3.37 ± 0.14	2.78 ± 0.15	2.69 ± 0.21 *	2.52 ± 0.17 **	2.14 ± 0.11 ***
Creatinine (mg/dl)	0.59 ± 0.02	2.34 ± 0.10 ***	2.57 ± 0.13 ***	2.53 ± 0.11 ***	3.19 ± 0.24 ***
Urea (mg/dl)	27.51 ± 1.72	55.87 ± 4.65 **	60.07 ± 2.86 **	58.46 ± 3.06 **	81.85 ± 5.99 ***

Data are mean ± standard error of the mean (SEM) (*n* = 6). * *p* < 0.05, ** *p* < 0.01, and *** *p* < 0.001 vs. control.

**Table 3:** Histopathological alterations in liver and kidney of MSNs-induced rats.

	Control	MSNs (mg/kg)			
		25	50	100	200
**Liver**					
Degenerative changes	-	++	++	+++	+++
Necrosis	-	++	++	+++	+++
Leukocyte infiltration	-	-	-	-	-
Hepatic parenchyma	-	-	-	-	-
Portal area	-	+	+	++	++
Congestion	-	+	++	++	++
Granulomatous reactions	-	-	-	+	+
Fatty changes	-	+	+	+	+
Kupffer cell activation	-	+	++	++	+++
**Kidney**					
Tubular degeneration	-	+	++	++	+++
Tubular necrosis	-	-	++	++	+++
Glomerulonephritis	-	+	+	++	+++
Glomerular atrophy	-	+	-	+	+++
Glomerular hypercellularity	-	+	++	++	++
Bowman’s capsule dilation	-	-	-	-	+
Leukocyte infiltration	-	++	+++	+++	+++
Chronic nephritis	-	+	++	++	+++
Others	-	-	Cast	Cast, edema, tubular dilation, cystic dilation	Cystic dilation, cast

## Supplementary Materials

The following are available online at https://www.mdpi.com/2218-273X/9/10/528/s1, Figure S1: Characterization of MSNs.; Methods: Synthesis and characterization of MSNs.
