# Supplementary material for: Mesoporous Silica Nanoparticles Trigger Liver and Kidney Injury and Fibrosis Via Altering TLR4/NF-κB, JAK2/STAT3 and Nrf2/HO-1 Signaling in Rats
Source: Biomolecules. 2019 Sep 25;9(10):528. doi: 10.3390/biom9100528 (PMC6843412; doi:10.3390/biom9100528)
Supplement: Supplementary file 1 [file biomolecules-09-00528-s001.pdf]

SUPPLEMENTARY MATERIAL

Mesoporous Silica Nanoparticles Trigger Liver and Kidney Injury and Fibrosis Via Altering TLR4/NF-κB, JAK2/STAT3 and Nrf2/HO-1 Signaling in Rats

Ayman M. Mahmoud [1,*], Ekram M. Desouky [2], Walaa G. Hozayen [2], May Bin-Jumah [3], El-Shaymaa El-Nahass [4], Hanan A. Soliman [2] and Ahmed A. Farghali [5]

1 Physiology Division, Zoology Department, Faculty of Science, Beni-Suef University, Beni-Suef 62514, Egypt
2 Biochemistry Department, Faculty of Science, Beni-Suef University, Beni-Suef 62514, Egypt
3 Department of Biology, College of Science, Princess Nourah bint Abdulrahman University, Riyadh 84428, Saudi Arabia
4 Department of Pathology, Faculty of Veterinary Medicine, Beni-Suef University, Beni-Suef 62514, Egypt
5 Materials Science and Nanotechnology Department, Faculty of Postgraduate Studies for Advanced Sciences (PSAS), Beni-Suef University, Beni-Suef 62514, Egypt
* Correspondence: ayman.mahmoud@science.bsu.edu.eg

Methods: Synthesis and Characterization of MSNs

The synthesis of MSNs has been conducted as previously described [1]. One g cetylpyridinium bromide and 0.6 g urea were dissolved in 30 ml deionized water followed by the addition of 30 ml cyclohexane and 1.2 ml isopropanol. 2.7 ml tetraethyl orthosilicate was added slowly and dropwise with strong stirring at room temperature (RT) and the mixture was heated to 85°C and incubated for 17 h, centrifugated, and washed with acetone and water. The synthesized MSNs were dried at RT for 12 h and calcined at 600°C for 6 h to remove the surfactant template. All chemicals were supplied by Sigma (St. Louis, MO, USA) and other standard suppliers. The synthesized material was characterized by scanning electron microscopy (SEM), transmission electron microscope (TEM), X-ray diffraction (XRD), zeta potential and thermal analysis as we reported recently [2].

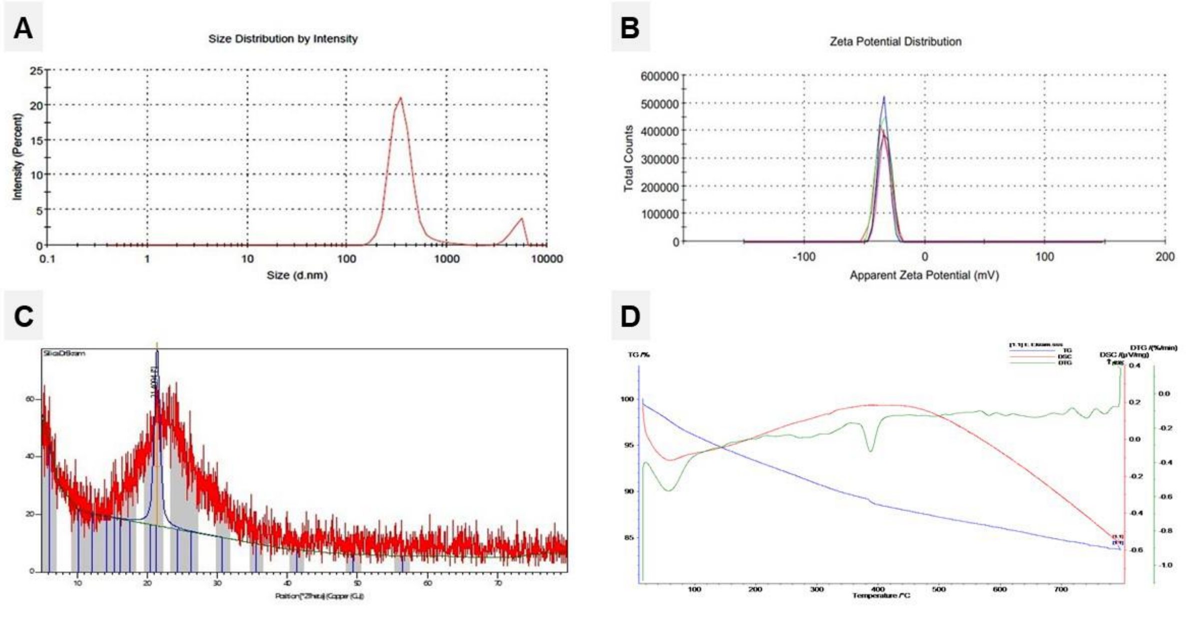

Supplementary Figure S1. Characterization of MSNs. (A) Dynamic light scattering (DLS) showing monodispersed sample preparation (pdi = 0.4), (B) Zeta potential showing a charge values about -35 mV, (C) X-ray diffraction pattern of MSNs showing the wide-angle XRD of KCC-1 at 21.4094º, and (D)

TGA/DSC profile showing weight loss features and two endothermic peaks at 100 and 400ºC, demonstrating water desorption and dehydroxylation of the surface, respectively. Reproduced with permission from [2].

References:

1. AbouAitah, K.; Swiderska-Sroda, A.; Farghali, A.A.; Wojnarowicz, J.; Stefanek, A.; Gierlotka, S.; Opalinska, A.; Allayeh, A.K.; Ciach, T.; Lojkowski, W. Folic acid-conjugated mesoporous silica particles as nanocarriers of natural prodrugs for cancer targeting and antioxidant action. *Oncotarget* **2018**, *9*, 26466–26490.
2. Hozayen, W.G.; Mahmoud, A.M.; Desouky, E.M.; El-Nahass, E.-S.; Soliman, H.A.; Farghali, A.A. Cardiac and pulmonary toxicity of mesoporous silica nanoparticles is associated with excessive ros production and redox imbalance in wistar rats. B*iomedicine & Pharmacotherapy* **2019**, *109*, 2527–2538.
